# In Living Color: Bacterial Pigments as an Untapped Resource in the Classroom and Beyond

**DOI:** 10.1371/journal.pbio.1000510

**Published:** 2010-10-05

**Authors:** Louise K. Charkoudian, Jay T. Fitzgerald, Chaitan Khosla, Andrea Champlin

**Affiliations:** 1 Stanford University, Stanford, California, United States of America; 2 School of the Museum of Fine Arts, Boston, Massachusetts, United States of America; University of California Berkeley/JGI, United States of America

## Introduction and Scope

Recent advances in the study of natural products made by bacteria have laid the foundation for engineering these molecules and for developing cost-effective ways to manufacture them. In our lab, we study a number of natural products that are synthesized by harmless soil bacteria of the *Streptomyces* genus. Whereas our primary interest in these molecules is due to their antibiotic properties, many of these natural products have distinct colors [1]. (The reasons for why *Streptomyces* make antibiotics or pigments remain mysterious.) This article is intended to make the case to the scientific and educational communities that *Streptomyces*-derived natural products are an untapped source of useful biopigments. By sharing some of our own experiences in harnessing these pigments to create paint and paintings, we also hope to inspire others to explore the potential of *Streptomyces*-derived pigments in art, industry, and perhaps most importantly, the classroom.

The pedagogical value of bacterial pigments is highlighted by the wide range of concepts and methods in chemistry, biology, and art that can be introduced to students in this context (see Box 1). Teachers can incorporate bacterial pigments into their lessons while introducing fundamental scientific principles ranging from the physics of color to the chemistry behind paints that fade in sunlight. Painting with living bacteria (Box 2) or extracting pigments from bacterial cultures (Box 3) provides a visual and kinesthetic activity to support key aspects of scientific investigations and methods learned in the classroom. Because the methods to do so are safe, inexpensive, and easily implementable in the everyday world, it is possible to use biopigments as a vehicle to introduce school children to science via art and vice versa. While many of these concepts and techniques are appropriate for the advanced high school or undergraduate classroom, even elementary school children can use bacterial paints prepared by their teacher to create art, an activity that may teach children at a young age that bacteria are a source of valuable materials rather than merely agents of disease.

**Box 1: Concepts at a Glance**

**Leads into chemistry, microbiology, and biotechnology**

- Chemical composition of paint (solubility and states of matter)^¥, ‡^
- Structures of pigment molecules (electromagnetic radiation, electron configuration, valence bonds, molecular orbital theory)^‡^
- Culturing *Streptomyces* and extracting their pigments (sterile culture techniques, natural product extraction techniques, solubility)^‡^
- Painting *Streptomyces* on agar plates (bacterial growth control)^¥,‡^
- Engineering bacteria to make new pigments (metabolic engineering of microbial systems)^‡^
- Scaling up the production of bacterial pigments (large scale bioprocessing techniques, recombinant DNA technology)^‡^
- UV absorber and radical scavengers as additives to paints (chemical structure and reactivity, radical reactions)^‡^

**Leads into fine arts**

- The perception of color (electromagnetic radiation, the eye as a spectrometer)^¥, ‡^
- Paint constituents (pigments, binders, solvents, surfactants, additives)^‡^
- Sources of pigments^‡^
- Making paints from pigments (grinding pigments, suspending in binder)^¥, ‡^
- History of pigments (art history)^*, ¥, ‡^

**Fun stuff**

- Drawing on paper with bacteria-derived paint^*, ¥, ‡^
- Creating living art by painting with bacteria on agar medium^*, ¥, ‡^

^‡^ = for undergraduate or advanced placement high school courses; ^¥^ = for high school courses; ^*^ = for elementary school courses

**Box 2: Living Art**

Since *Streptomyces* spores spread over a limited distance when cultured on semi-solid agar medium, the site of inoculation of a petri-dish will determine the boundaries of bacterial growth [1]. Thus, the spores of *Streptomyces* can be used as paint, and the artist's brush strokes will dictate the final image (for example, see Image 1).

**Figure:**
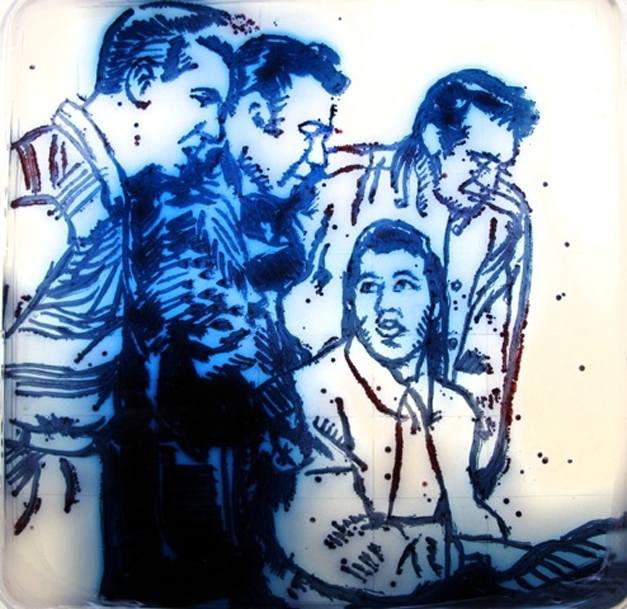
Image 1. “Elvis Lives!” painted on R5 media plates using *S. coelicolor*.

Prior to inoculating the petri dish (see Box 3 for agar recipe) with *Streptomyces* spores, freshly sterilize the surface of a designated area and the bristles of the paintbrush(es) using rubbing alcohol. Once the paintbrush bristles have dried, the tip of the brush can be used to gently lift spores from a source (typically another petri dish with a lawn of well-sporulated bacteria), and applied to a fresh petri dish as “paint.” The transfer of spores will appear as a colorless residue on the media surface. Since nutrient medium in a petri dish is moderately translucent, an image can be placed under the dish as a template for tracing. During the painting process, if a brush stroke is made in error, a paintbrush dipped in rubbing alcohol can be used as an eraser. If multiple strains are used to produce artwork composed of multiple colors, a separate paintbrush should be designated for each strain to avoid contamination. Notably, since *Streptomyces* produce antibiotics along with their pigments, overlaying different strains may result in competitive growth (i.e., one bacterial strain may be sensitive to the other strain's antibiotic). This can sometimes present a challenge, but also an opportunity.

Once an image is complete, the petri dish is covered with its lid and placed bottom-side up for incubation. *Streptomyces* will grow fastest at 30 °C, usually showing pigmentation in 2–3 days. At room temperature, strains will grow more slowly and will likely produce pigment after 7–10 days. When the painting has developed to a desired pigmented state, the petri dish can be stored in a refrigerator. Though the agar in the petri dish will continue to dry slowly at the cooler temperatures, the drying rate can be reduced by wrapping the edge of the dish with tape.

**Box 3: Isolation of Pigments from *Streptomyces***

Normally, *Streptomyces* strains are cultured on sterilized complex semi-solid medium containing yeast extract such as R5 media; this ensures abundant sporulation [1]. However, *Streptomyces* are nutritionally quite versatile and are capable of utilizing a wide variety of carbon sources. In fact, the *Streptomyces* strains evaluated in our lab were easily cultured on a semi-solid medium made simply with chicken broth and agar, making it possible to culture pigment-producing strains at home or in the classroom. Prior to making the nutrient plates, a designated area and all containers should be thoroughly disinfected with rubbing alcohol or Lysol. To make the media, add ∼4 g agar (2 teaspoons) to 230 mL (1 cup) chicken broth and sterilize using an autoclave, stovetop, or a pressure cooker. After sterilization, allow the solution to cool to ∼50 °C prior to pouring into petri dishes (20 mL/dish). Once the broth solidifies and looks relatively dry, the plates can be used immediately or covered and stored in a refrigerator for future use.

To obtain a confluent culture (“lawn”) of *Streptomyces* on the petri dish, bacteria should be inoculated over the entire surface of the medium using broad strokes. The petri dish is then covered with its top and placed bottom-side up for incubation. *Streptomyces* will grow fastest at 30 °C, usually showing pigmentation in 2–3 days. At room temperature, strains will grow more slowly, and will likely display pigmentation after 7–10 days. When the culture has developed to a saturated pigmented state, the pigment can be extracted from the media.

Red and blue paint can be easily made from the extracts of *S. coelicolor*. Actinorhodin is water-soluble and can be readily extracted from a confluent culture by breaking up the agar into small pieces (use a spoon or spatula to obtain pieces of ∼1 cm^3^) and adding a minimal amount of water (∼15 mL per petri dish). Within minutes, the pigment will leach out of the agar creating a blue aqueous solution. At this point, the solution can be separated from the agar by soaking it up with a cotton ball (using forceps) and releasing the solution into a clean petri dish. To obtain a red pigment, titrate hydrochloric acid (or vinegar) into an aliquot of the blue actinorhodin solution until a red color stabilizes. The pigmented solutions can be left in an open petri dish to air dry.

## A Brief Introduction to Paint

Commercial paints have many ingredients, including pigments, binders, solvents, surfactants and/or other additives. The chemical properties of these substances dictate the practical characteristics of paints. In both art and industry, the recipe for a given paint is judiciously developed from available ingredients to optimize color, ease of use, and stability. The binder creates a solid, tack-free film, which solidifies upon curing, evaporation, or cooling. Popular binders include oils, alkyds, eggs, acrylics, gums, and waxes. Solvents are added to facilitate application, expedite drying, and increase the homogeneity of the paint film. They are temporary components of paint, evaporating evenly and totally as the paint dries. The addition of a surfactant decreases the surface tension of the liquid allowing for easier application of the paint to a surface.

The centerpiece of each paint is a dye or pigment molecule that combines with the colorless glaze generated by the other ingredients to produce a vibrantly colored material. Dyes and pigments contain chemical functional groups capable of delocalizing electrons. When visible light (which comprises of electromagnetic radiation of wavelengths in the 400–780 nm range) strikes these molecules, they absorb radiation at a specific wavelength. The human eye then detects those wavelengths that are not absorbed by the dye or pigment, and the brain perceives color from this visual information. The fundamental difference between dyes and pigments lies in the solubility of the colored molecule in the medium in which it is dispersed. Whereas dye molecules are soluble, pigments are insoluble and exist as distinct particles suspended in the binder.

## Sources of Pigments

Conventional sources of pigments range from inorganic metals and metal oxides to organic molecules. Paleolithic humans made paint 30,000 years ago by grinding up earth pigments such as red ochre, which gets its color from hematite (Fe_2_O_3_), yellow ochre (Fe_2_O_3_·H_2_O), and green malachite (Cu_2_CO_3_(OH)_2_) and suspending these in their own saliva as a binder [2]. These prehistoric pigments carved out a place on the modern artist's palette and have been joined by other common inorganic pigments including titanium white (TiO_2_), cobalt blue (CuO + Al_2_O_3_), Egyptian blue (CuO•CaO•SiO_2_), cadmium red (CdSe), and black oxide of iron (FeO). The source of delocalized electrons in these pigments is the metal–ligand complex.

**Figure 1:**
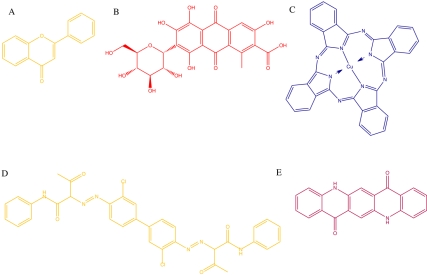
Structures of natural and synthetic pigments. (A) flavone, a yellow pigment produced by paints; (B) carminic acid, a red pigment produced by scale insects; (C) copper phthalocyanine-blue, a blue pigment of the synthetic phthalocyanine family; (D) benzidine yellow, an azo pigment of the synthetic arylide family; (E) PV19, a red pigment of the synthetic quinacridone family. Each molecule is colored to match its perceived color.

Traditional sources of organic pigments and dyes include natural products such as flavinoids (Figure 1, compound A) and anthraquinones (Figure 1, compound B) produced by plants and animals. For example, carminic acid, a deep red anthraquinone produced by scale insects, is now used as a pigment in paints, crimson ink, cosmetics, and food colors [3]. Over the course of the 20th century, naturally occurring organic pigments have been almost completely displaced by synthetic molecules such as phthalocyanines that range from blue to green (Figure 1, compound C), arylides that are yellow to greenish- or reddish-yellow (Figure 1, compound D), and quinacridones, ranging from orange to violet (Figure 1, compound E) [4]. Advances in organic chemistry enabled mass production of these compounds relatively cheaply, thereby allowing them to displace natural product pigments, whose procurement is often more challenging.

**Figure 2:**
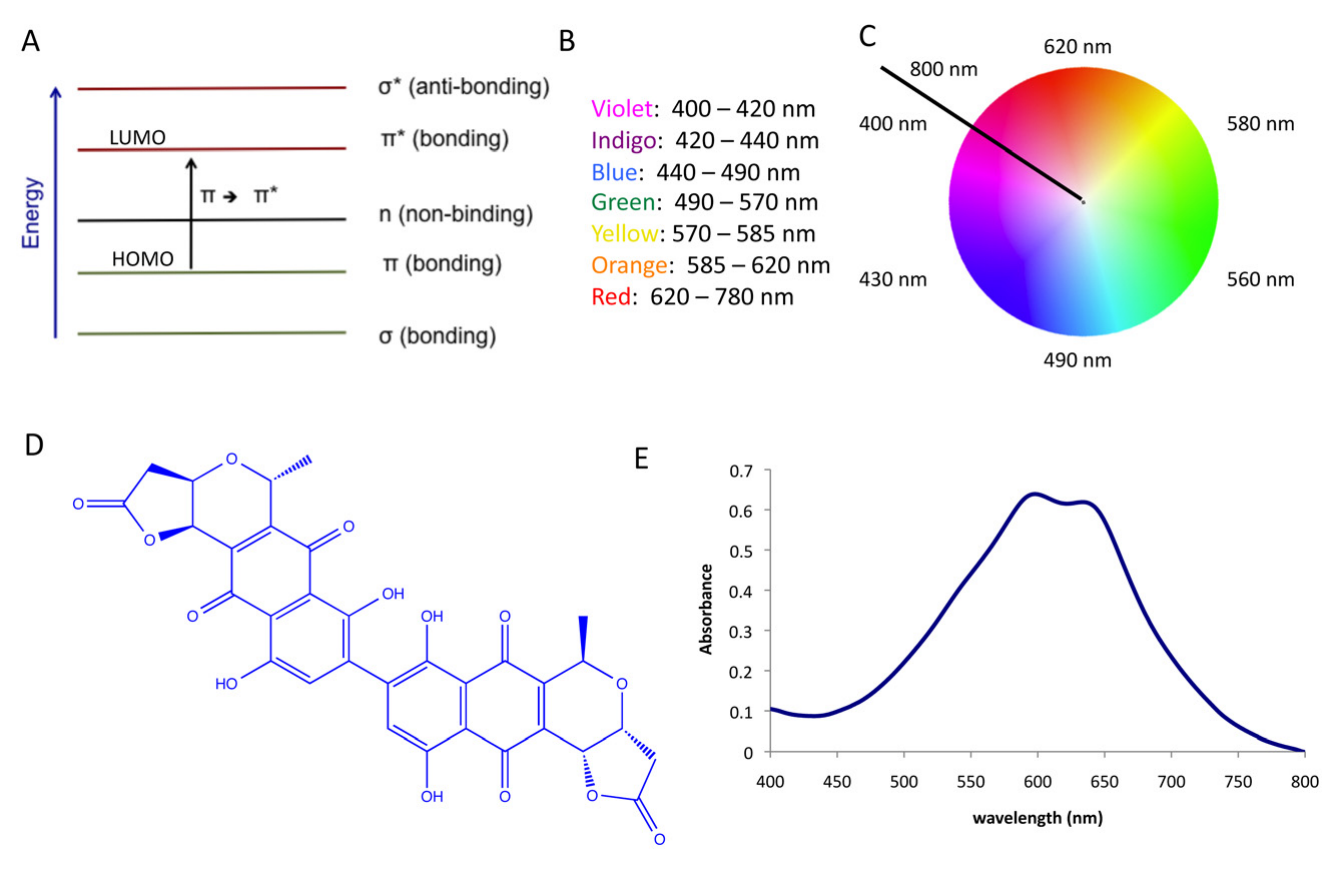
Using the biopigment actinorhodin as a case study to understand the logic of color. (A) When light strikes a pigment, electrons absorb energy and are transferred from the HOMO to LUMO; (B) Relationship between wavelength of visible light and its color; (C) The complementary relationship between color of the absorbed light and the perceived color; (D) Structure of actinorhodin; (E) Absorption spectrum of actinorhodin. The pigment absorbs light at 600 nm, and is therefore perceived as blue by the brain.

The color of organic dyes and pigments is the result of a network of conjugated π electrons that delocalize over a large portion of the molecule (Figure 2). This network causes the molecule to have a well-defined energy gap between the highest occupied molecular orbital (HOMO) and lowest unoccupied molecular orbital (LUMO). The magnitude of this energy gap dictates the wavelength of visible light that will be absorbed by the dye or pigment (Figure 2A). The remaining light is reflected, and will assume the complementary color to the wavelength(s) absorbed (Figure 2B and C). Thus, the bacterial pigment actinorhodin (Figure 2D), which absorbs light at 600 nm (Figure 2E), is perceived as blue by the brain.

## Bacteria and Their Pigments

**Figure 3:**
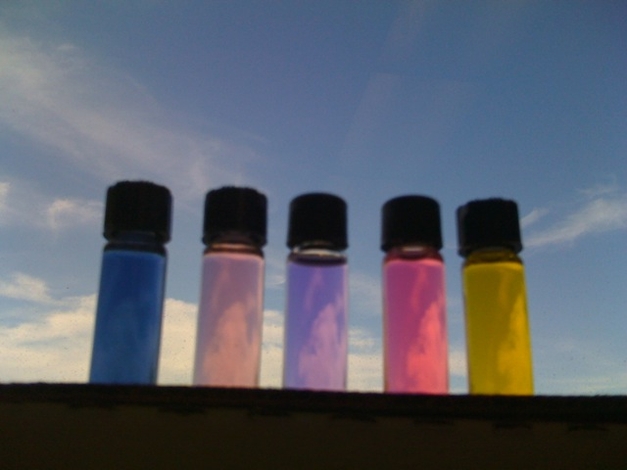
Extracts from *Streptomyces* cultured on semi-solid agar plates. *S. coelicolor* extracted with water (A) and after addition of dilute acid (B); *S. violaceoruber* Tu22 extracted with ethyl acetate (C) and after addition of dilute hydrochloric acid (D); *S. roseofulvus* extracted with ethyl acetate (E).

Bacteria of the genus *Streptomyces* produce important antibiotics such as streptomycin and tetracycline [1]. They also produce many intensely pigmented molecules that can be isolated in a pure form. In particular, natural products called polyketides include pigments that span the visible light spectrum (Figure 3). For example, *Streptomyces coelicolor* produces the ultramarine blue pigment actinorhodin (Figure 1D). Each *Streptomyces* strain releases its own distinct cocktail of pigmented natural products that determines the strain's characteristic leached hue. Some pigments, such as actinorhodin, change color in a pH-dependent manner (see, for example, Figure 3) [1], whereas others maintain their characteristic color over a range of pH.

**Figure 4:**
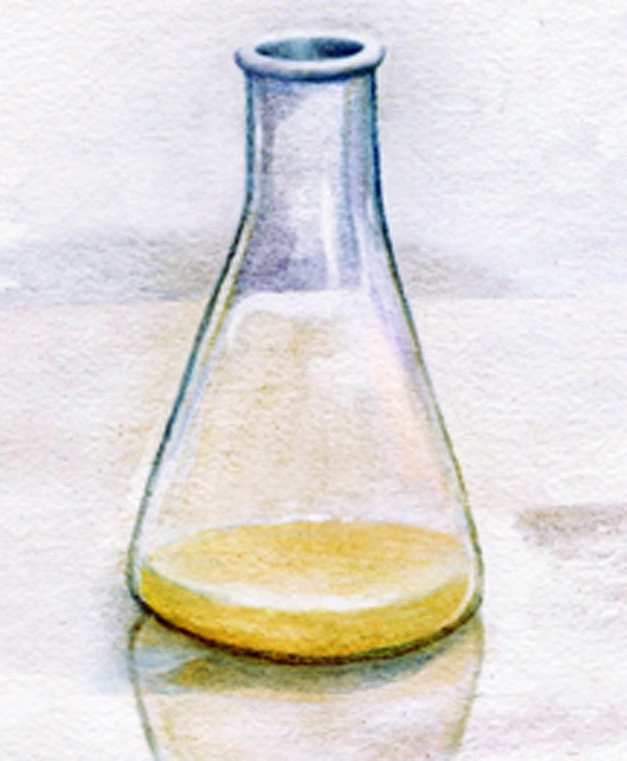
Painting produced exclusively with biopigments extracted from *Streptomyces* and suspended in acrylic binder.

The ability of *Streptomyces* to produce pigments when propagated on semi-solid medium can be exploited to create living art (Box 2). Alternatively, the excreted pigments can be isolated from *Streptomyces* strains easily cultured in a classroom or kitchen setting [5] (Box 3). To make paint from *Streptomyces* pigments, the dried, colored extract must be resuspended in a binder. In our experience, *Streptomyces* pigments are extremely compatible with acrylics, which have emerged as the predominant class of paint binders in the latter half of the 20th century. To make a homogeneous paint, the pigment should be finely ground prior to the addition of a minimal amount of acrylic binder. Solvents (e.g., water for acrylic paints or petroleum distillates for oil-based paints) can then be added to optimize the application properties of the paint. Figure 4 shows an example of a painting produced with four different pigments extracted from *Streptomyces* and suspended in acrylic binder.

## Large-Scale Production of Designer Bacterial Pigments

Whereas small quantities of pigment are sufficient for making paints used in an artist's studio or a classroom, massive quantities of pigments are required for industrial applications. Therefore, the utility of a pigment is dictated not only by its inherent properties but also by the ability to produce it in sufficient quantities. Although there are several challenges associated with scaling up pigment production and isolation from *Streptomyces* bacteria, much of the technology to overcome these challenges is already in place, which provides a potential route for reintroducing biopigments to a cost-sensitive world. For example, the pragmatic constraint of culturing a large number of semi-solid media plates, which would require the use of many petri-dishes as well as large incubators, can be overcome using fermentation tanks (much like those in a brewery). In fact, such fermentation technology is already used to produce natural products from *Streptomyces* for pharmaceutical, animal health, and agricultural applications. A more significant challenge lies in the need to increase the production of the bacterial pigment from a given *Streptomyces* strain to make its manufacture economically viable. Here, recent developments in molecular biology could be of use. The genes responsible for the biosynthesis of numerous pigments have been cloned, and recombinant DNA technology has been harnessed to overproduce these pigments [6],[7]. For example, scientists at Amgen, Inc. were able to engineer a widely used non-hazardous strain of *Escherichia coli* to overproduce indigo (which at one point was exclusively derived from the woad plant) in fermentation tanks [8],.

Biosynthetic pathways can also be manipulated to engineer a pigment's molecular structure and consequently its color. For example, *Streptomyces coelicolor*, which produces the blue pigment actinorhodin (Figure 2, compound D), can be genetically modified to produce a related polyketide called kalafungin, which is bright yellow [10]. Alternatively, actinorhodin biosynthesis can also be engineered to produce orange or yellow-red anthraquinones [11],[12].

Yet another challenge involves isolation of these pigments in relatively pure and concentrated forms. Broadly speaking, *Streptomyces* produce two types of pigments—those that predominantly remain bound to the bacterial mycelia and those that are secreted into the fermentation broth. Whereas pigments from the former class can be conveniently recovered by disrupting the filtered mycelia with acetone, secreted natural products are typically recovered by extracting the aqueous broth with large quantities of organic solvents such as ethyl acetate. To mitigate environmental and health concerns associated with solvent use, alternative separation technologies such as spray-drying (widespread in the food and feed industry) and solid-phase extraction (commonplace in the fine chemical industry) may be appropriate.

Last but not least, to be useful in paint, a pigment must have acceptable stability when exposed to environmental stresses, especially UV light. UV light initiates undesirable free-radical reactions in paints that ultimately lead to their degradation. A variety of UV absorbers (such as benzotriazole- and triazine-based molecules) and free-radical scavengers (such as hindered amines) are already used in the paint industry and are commercially available. Whereas their effectiveness in conjunction with biopigments remains to be studied, they have the potential to enhance the utility of *Streptomyces*-derived natural products in paints.

## Concluding Remarks

Soil bacteria from the *Streptomyces* genus represent a source of interesting natural products that have been largely overlooked by artists, researchers, and teachers. This article is intended to encourage amateurs and professionals alike to explore this overflowing source of biopigments. Not only does this endeavor have the potential to lead us toward a fertile nexus between art and science, it may also lead to a more sustainable and environmentally friendly way to color the world around us in the future. The relevance of biopigments to many facets of science, technology, and society, makes this material an outstanding tool to engage students of varying academic interests across multiple age groups. Therefore, we encourage teachers of all levels to consider using biopigments as a vehicle to introduce the scientific method to their students. To facilitate the implementation of biopigments into science and art curricula, we have provided a list of useful online resources and information about procuring materials (see Box 4) as well as recommend ways to evaluate the effectiveness of the lesson (see Box 5).

**Box 4: Teaching Tools**

**Useful online resources for background information**

- Pigments and paints: http://www.webexhibits.org/pigments/
- Visible and ultraviolet spectroscopy: http://www2.chemistry.msu.edu:80/faculty/reusch/VirtTxtJml/Spectrpy/UV-Vis/spectrum.htm
- http://science.hq.nasa.gov/kids/imagers/ems/visible.html
- *Streptomyces* microbiology:
- http://openwetware.org/wiki/Streptomyces:Other_Bits/An_Introduction_to_Streptomyces
- Recombinant DNA technology: http://webapps.css.udel.edu/biotech/rDNA.html

**Shopping resources**

- Agar: Health food stores (Whole Foods), Asian grocers, home science tools (http://www.hometrainingtools.com)
- Petri dishes: home science tools (http://www.hometrainingtools.com)
- Disinfectants: drugstores, hardware stores
- *Streptomyces* strains: ATCC (www.atcc.org)
- Acrylic binder: art suppliers, Golden acrylic (http://www.goldenpaints.com/)

**Box 5: Evaluation Tools**

**Questions to gauge interest and knowledge before the lesson**

- What do you think of when you think of bacteria?
- Why do we need to sterilize our tools before growing bacteria?
- What do bacteria need to grow?
- What is a natural product?
- Why are objects colored?
- How are paints made?
- Why do paints fade over time?

**Questions to gauge understanding after the lesson**

- What are some ways in which bacteria can be useful to humans?
- How do bacteria produce useful natural products?
  - How can we manipulate bacteria to create new products?
- What are some sources of natural products and how can we harness them?
  - Why do some pigments prefer organic solvents to water?
- What makes us perceive color?
  - Why are some molecules colored and some are not?
  - How can we use the color wheel to predict an object's color from its absorption spectrum?
- How can we prevent paints from fading over time?

**Evaluative activities**

- Have students present their projects and discuss what their initial plans were, what could have been improved along the way, and what things surprised them about working with bacteria and pigments.
- Have students research an aspect of paint production, color, or microbiology and present their findings to the class.
